# An Auction-Based Spectrum Leasing Mechanism for Mobile Macro-Femtocell Networks of IoT

**DOI:** 10.3390/s17020380

**Published:** 2017-02-16

**Authors:** Xin Chen, Lei Xing, Tie Qiu, Zhuo Li

**Affiliations:** 1 School of Computer Science, Beijing Information Science and Technology University, Beijing 100101, China; chenxin@bistu.edu.cn (X.C.); xinglei@mail.bistu.edu.cn (L.X.); lizhuo@bistu.edu.cn (Z.L.); 2 School of Software, Dalian University of Technology, Dalian 116620, China

**Keywords:** Internet of Things, spectrum leasing, pricing framework, auction, knapsack problem

## Abstract

The Internet of Things (IoT) is a vision of the upcoming society. It can provide pervasive communication between two or more entities using 4G-LTE (Long Term Evolution) communication technology. In 4G-LTE networks, there are two important problems: helping manage the spectrum demands of IoT devices and achieving much more revenue with the limited resource. This paper proposes a pricing framework to investigate the spectrum leasing in mobile heterogeneous networks with single macrocell and multiple femtocells. We modeled the leasing procedure between the macrocell service provider (MSP) and femtocell holders (FHs) as an auction to motivate the MSP to lease its spectrum resource. All FHs act as the bidders, and the monopolist MSP acts as the auctioneer. In the auction, FHs submit their bids to rent the spectrum resource so that they can make a profit by selling it to their subscribers. The MSP determines the spectrum leasing amount and chooses the winning FHs by solving the dynamic programming-based 0–1 knapsack problem. In our proposed framework, we focus on the spectrum pricing strategy and revenue maximization of the MSP. The simulation results show that the proposed scheme provides effective motivation for the MSP to lease the spectrum to FHs, and both the MSP and FHs can benefit from spectrum leasing.

## 1. Introduction

In recent years, with the rapid development of the Internet of Things (IoT) [1] and cloud computing [2], the number of user equipment (UE) accessing mobile networks is growing quickly, resulting in the increasing of network traffic. The spectrum available will be extremely crowded in the near future [3]. The emerging wireless communication systems will have to support, besides data traffic generated by human, also machine-generated traffic [4]. As a new information acquisition system, IoT contains multiple types of heterogeneous networks, and it has been widely used in many applications where the scale of the sensory data has already exceeded several petabytes annually [1,5]. The next generation of mobile networks, i.e., 5G, is set to play an important role in supporting these applications [6]. IoT enables objects to collect or exchange data by using many network technologies, such as wireless sensor networks (WSN), wireless communication and data collection [7]. Among them, WSN consists of a set of physically small sensor nodes deployed in the monitoring area and plays an irreplaceable role in IoT due to its self-organization, good concealment and high fault tolerance [8,9,10]. With the increasing scale and devices update, the proliferation of wireless devices in industrial applications makes the spectrum sharing in limited ISM (industrial, scientific and medical) become a challenging problem [11]. Meanwhile, limited spectrum resources and an inflexible spectrum assignment policy bring about poor spectrum utilization currently [12].

In 4G LTE-Advanced networks, there are many low-cost and low-power wireless access points (WAPs), which are randomly distributed. Those WAPs provide data access services for a variety of wireless devices. These services enable wireless devices around us, such as smart phones, sensors, tablet computers and other equipment, to communicate with each other. This is also the basic idea of IoT [13]. Therefore, spectrum demand is increasing rapidly in 4G networks. Furthermore, the demand of the spectrum resource is expected to increase significantly in 5G networks [14]. Because of its scarcity, the spectrum is one of the most precious resources in many wireless networks [15,16,17], such as wireless mesh networks [18], industrial wireless sensor networks [19] and underwater sensor networks [20]. Meanwhile, the secure communication, energy efficiency and spectrum efficiency of the wireless networks are also being discussed widely. As a hot topic, security has become a challenging problem in various fields [21]. Additionally, in [22,23,24], the authors deeply studied the physical layer security of secure communication in wireless networks. Furthermore, the energy issue has been discussed in many existing works, such as [25,26]. For mobile operators to provide communication services, the spectrum can bring out much more revenue with the rapid growth of demand from spectrum users. In the mobile macro-femtocell networks, macrocell base stations (MBSs) are deployed by the macrocell service provider (MSP) and femtocell base stations (FBSs) by large pool of femtocell holders (FHs). The MBSs own all of the licensed spectrum and make a profit from macrocell UEs (MUEs) by selling spectrum bandwidth as the resource holders. However, severe signal attenuation at these high frequencies often causes poor signal reception for indoor users, who are separated by walls from outdoor MBSs , which causes low spectrum efficiency and results in insufficient resource utilization.

The idea of the femtocell has been proposed to solve the poor signal reception problem for indoor users, contributing towards the improvement of coverage and throughput. Generally, femtocells are comprised of small-sized, low-power, low-cost and short-range access points, which shortens the distance between base stations and UEs, thereby offering better quality of service (QoS) [27,28]. In general, femtocells are deployed to run in one of the three access modes: closed access, open access and hybrid access.

- In closed access mode, only a subset of users who have been authorized by the FH can access FBS. This access mode avoids undesired traffic congestion and provides effective privacy protection for authorized users.
- Open access allows all customers of the operator to access FBS. However, the QoS of the FBS subscribers may degrade if the FBS resources are utilized for non-subscribers.
- Hybrid access mode, which is the mixture of the two, can protect the registered users and shows the most potential for entire network performance enhancement.

We will focus on the closed access mode in this paper, because it is more conducive to the protection of privacy than the other two access modes.

However, the FHs own no spectrum and expect to rent some spectrum from the MSP for serving their subscribers, so that it is a meaningful issue to investigate the spectrum leasing between the MSP and FHs. For the MSP, its main consideration is how to improve the spectrum efficiency to increase the benefits. For the FHs, their concern is how to get spectrum resources from the MSP. The main challenges include: (1) how to motivate the MSP to lease spectrum to the FHs and how to determine the spectrum leasing amount; (2) how to determine the spectrum leasing price that both the MSP and FHs can accept.

In order to answer these questions above, we propose a spectrum leasing framework based on auction. In the view of economics, auctions are an effective way to investigate resource allocation problems in wireless communications [29]. In our proposed framework, we focus on the spectrum pricing strategy and revenue maximization of the MSP. Meanwhile, the overall network throughput has been improved. The main contributions of this paper are as follows:

- We propose a two-tier network pricing framework to investigate the revenue maximization problem of spectrum leasing in the heterogeneous networks with single macrocell and multiple femtocells. Unlike most of previous works, which focus on enhancing the throughput or capacity, the proposed framework mainly considers the pricing strategy for operator to obtain maximal profit.
- We model the spectrum leasing procedure between the MSP and FHs as an auction, where the monopolist MBS is the auctioneer and all FBSs the bidders. We focus on a certain action of each bidder, which means that each FBS is independent of the others. The amount of resources leased to femtocells from the MSP is optimized with pricing parameters to filter those MUEs with a bad channel condition.
- In our model, price is not the only decisive factor, so that bidders have no motivation to lie for winning. The auction results are determined by both price and bandwidth. The price and bandwidth can be mapped as the value and weight of the knapsack problem. Therefore, we introduce the concept of the knapsack problem and design a dynamic programming-based algorithm to solve the combinatorial optimization problem.
- We conduct extensive experiments to simulate the real network environment and verify the effectiveness of our proposed mechanism. The simulation results show that the proposed framework provides effective motivation for the MSP to lease spectrum to FHs and improves the networks’ throughput. Additionally, both the MSP and FHs can achieve higher utility with spectrum leasing.

The rest of this paper is organized as follows. Section 2 discusses the studies related to the proposed framework. We introduce the system model, including the scenario and channel model in Section 3. In Section 4, we formulate the problem as a revenue function. In Section 5, we explain the auction procedure, which is played in two phases. The simulation results are shown in Section 6, and we give the conclusion in Section 7.

## 2. Related Work

Many academic researchers are committed to the research of related issues in the femtocell domain. Most previous works on femtocells focused on the resource allocation issue [15,17,27,28,30,31,32,33,34,35,36]. In [15,28], the authors considered imperfect channel state information in the resource allocation of cognitive networks, which is more reasonable in realistic scenarios. In [17,27], different resource allocation schemes were proposed to maximize the capacity of the system under the interference constraint. The difference is that we considered the economic factor in the proposed spectrum allocation scheme.

Game theory is a common method to present a competitive relationship. In [30], the authors studied pricing issues for femtocell service using a game theoretic approach. A leader follower game and a non-cooperative simultaneous game are adopted in the monopoly and duopoly cellular service markets, respectively. The authors in [31] proposed a dynamic hybrid access algorithm to provide incentives to the FBS for sharing its resources with unregistered users. The Stackelberg game model was adopted to represent the competition behaviors of the FBS and users. However, the MBS does not participate in the game. The authors of [32] investigated the joint subchannel and power allocation problem using cooperative Nash bargaining game theory, and uplink is considered.

Auctions are an effective method to model the resource allocation problems. In [33,34], the authors studied two different Vickrey-Clarke-Groves (VCG)-based auction mechanisms to motivate private femtocells to serve unregistered users. Additionally, in [35], the authors investigated a resource allocation scheme in the uplink transmission for enhancing each femtocell user and the whole macrocell network’s revenue, both by introducing the auction mechanism. In [36], the authors proposed a spectrum sharing framework for motivating hybrid access in two-tier macro-femtocell networks. They designed an auction mechanism to allocate spectrum resources. The auction results in a win-win solution since both utilities of the MBS and FBSs are maximized, which attracted us to study the auction method to solve our problems. In that model, the objective of the FBSs is to gain revenue by selling their free spectrum to serve the nearby MUEs. However, we consider a scenario where FBSs own no spectrum, and the objective of FBSs is to obtain the spectrum from the MSP for serving their subscribers.

Only a few papers have studied the pricing strategies of the spectrum leasing problem [37,38,39,40]. In [37], the authors proposed a framework towards uplink macrocell and femtocell cooperation under a closed access policy, in which a femtocell user may act as a relay for macrocell users and each cooperative MUE grants the femtocell user a fraction of its superframe. However, downlink transmission is not considered in the paper.

In [38], the authors proposed a spectrum leasing framework taking hybrid access into consideration. A three-stage Stackelberg game is proposed to model the procedure between the MSP and FH. This game provides sufficient incentive for their cooperation, and both the MSP and FH can benefit from spectrum leasing. In the view of energy efficiency, a cooperative spectrum leasing framework is proposed to both mitigate interference and save energy in [39], and the authors also formulated the spectrum leasing procedure as a Stackelberg game.

In [40], the authors proposed an economic incentive and analyzed the economic impacts of adding femtocell service on the revenue of the operator. They modeled the interactions between operator and users as a Stackelberg game, which inspired our work. In [41], an optimal service auction was proposed, which allows users to access an IoT device. The example they discussed provides strong support for our work in the IoT case.

According to the analysis above, the previous works often use game theory, especially the Stackelberg game, to model the spectrum leasing problem. Stackelberg game players are a leader and a follower. The leader moves first and the follower subsequently. In two-tier femtocell networks, the follower of all of the FBSs, as a whole, has consistent behaviors. Additionally, the leader MBS must know ex-ante that the follower observes its action before it decides. However, we focus on the certain action of each FBS, which means that each FBS is independent of the others. The MBS makes a decision without considering the action of FBSs. Therefore, we propose a spectrum leasing framework based on auction to investigate how to allocate resources to each FBS. From the perspective of the economy, auctions are a powerful tool to model, analyze and optimize the spectrum leasing problem when multiple femtocells exist in the heterogeneous networks. In our model, the spectrum is limited, and all users who want to obtain spectrum resources have to participate in the competition, which ensures the fair allocation of resources and the income maximization of the operator.

## 3. System Model

### 3.1. Scenario

We consider a two-tier macro-femto network based on the auction mechanism. The scenario contains one MBS in the center, which belongs to a MSP, and *K* FBSs distributed randomly, which belong to *K* FHs and *L* MUEs. The MSP is a traditional wireless service provider that provides data access service to its MUEs with MBS, while the FH is a fixed-line service provider that provides wireless access service to indoor users with FBS. The MSP is the spectrum license holder who owns all of the available spectrum in the networks. It is assumed that the MBS sells spectrum resources to its MUEs with a unified price. However, the FHs own no spectrum and have to rent spectrum from the MSP according to the price charged by the MSP when they want to serve their subscribers, i.e., femtocell UEs (FUEs), for making a profit. In our scenario, all MUEs are assumed to be uniformly distributed inside the cell site.

**Figure 1:**
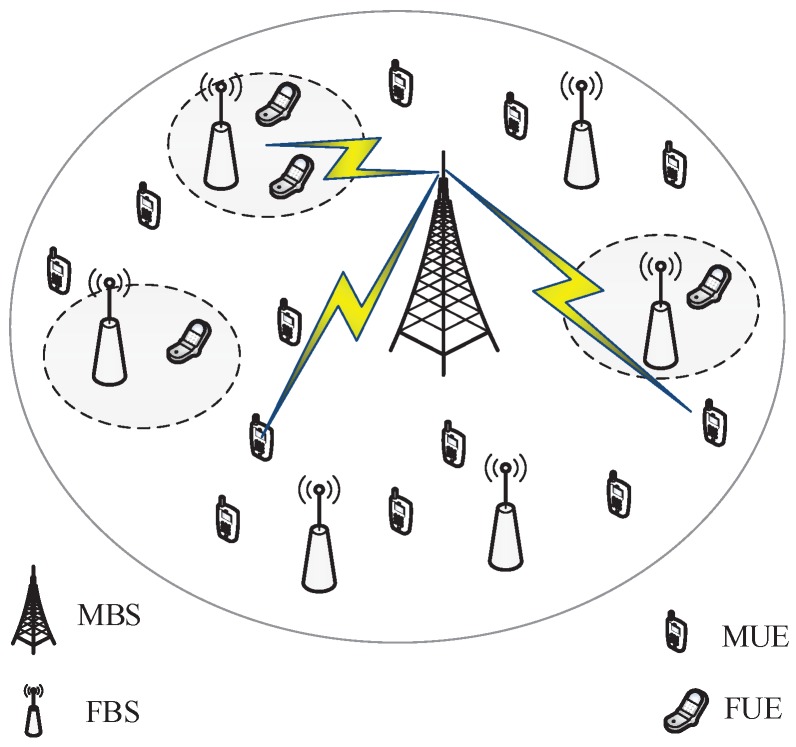
Two-tier macro-femto networks architecture.

The MBS owns a total *W* Hz wireless spectrum bandwidth to provide macrocell data access service, where each MUE is allocated part of the bandwidth by purchasing from MBS. Figure 1 presents the two-tier macro-femto network architecture. As shown in Figure 1, there are *K* FBSs and *L* MUEs within the coverage of the MBS, denoted as ${\left\{F_{i}\right\}}_{i=1}^{K}$ and ${\left\{MU_{i}\right\}}_{i=1}^{L}$, respectively. In this paper, we will study the optimal pricing strategy $\left(\gamma_{U,M},\gamma_{U,F},l_{F,M}\right)$, where $\gamma_{U,M}$ is the macrocell service price per unit bandwidth, determined by the MSP, $\gamma_{U,F}$ is the femtocell service price per unit bandwidth, determined by FHs, and $l_{F,M}$ is the spectrum leasing price per unit bandwidth, submitted by FBSs.

**Figure 2:**
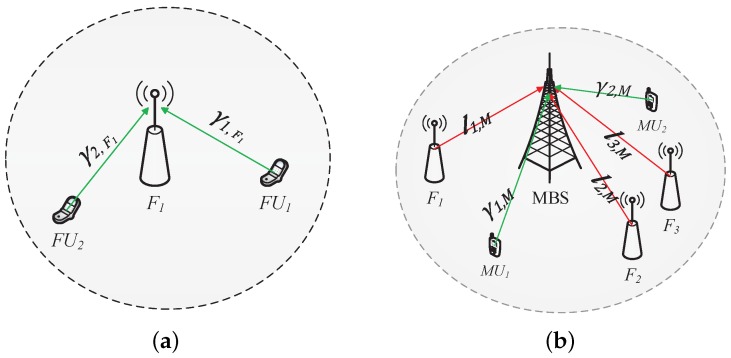
Auction-based pricing procedure for spectrum leasing. (**a**) femtocell base station (FBS) service price determination; (**b**) macrocell base station (MBS) service price and spectrum leasing price determination.

We present the general procedure of spectrum leasing in Figure 2. The auction procedure is played in two phases. Phase 1 is shown in Figure 2a; FBSs optimize their pricing strategies independently of each other. In the figure, the $F_{1}$ determines service price $\gamma_{1,F_{1}}$, $\gamma_{2,F_{1}}$ for its subscribers $FU_{1}$, $FU_{2}$ and calculates the optimal rent price. Then, in the second phase, as shown in Figure 2b, the MBS first announces its service price $\gamma_{1,M}$, $\gamma_{2,M}$, where $\gamma_{1,M}$ = $\gamma_{2,M}$, and charges service fees from its served MUEs. Meanwhile, all FBSs submit their spectrum renting price $l_{1,M}$, $l_{2,M}$, $l_{3,M}$ and note that the three of them are not necessarily equal. Then, the MBS acts as the auctioneer who receives the bids from all of the FBSs and determines the winner FBSs by optimizing its own revenue. Finally, the MBS charges leasing fees from those winner FBSs and leases corresponding spectrum resources to them. We will discuss the details in Section 5.

#### IoT Scenario

**Figure 3:**
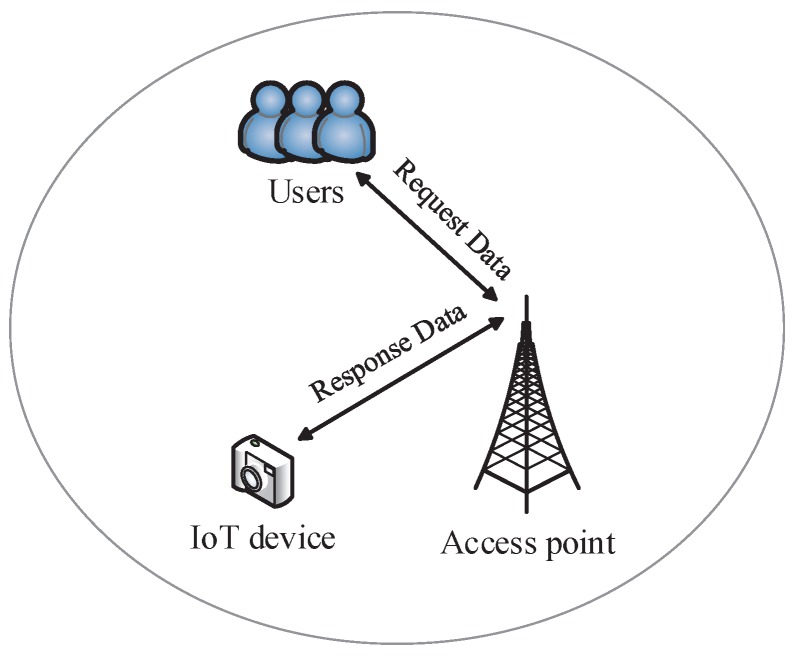
IoT scenario of spectrum leasing.

To illustrate the applicability of our proposed spectrum sharing approach in the IoT scenario, we consider an IoT device that can be allocated to serve an online user, for example a camera, as [41] mentioned. As shown in Figure 3, there are some online users who request to control the device. After the device receives the requested data, it firstly determines which user obtains the right of control, and then, it will transmit the result to an access point; and the access point will forward the data to those users subsequently. Finally, the connection between the winning user and the device will be established. In this process, as a spectrum user, the IoT device has to pay for using spectrum resources. Additionally, the cost will be on the basis of the charges to provide service for the winning user. Therefore, the proposed scheme can be used in the IoT scenario.

### 3.2. Channel Model

We consider the closed-access downlink transmission in the given network scenario. In order to simplify the model and avoid complex cell interferences, we assume different femtocells and macrocell are using non-overlapping frequency bands. Therefore, there is no cross-tier interference or intra-tier interference in the overall network. The background noises accounting for the additional interference from other cells at the users are assumed to be independently and identically distributed complex Gaussian random variables with zero mean and a common variance $\sigma^{2}$ [31]. It is assumed that the background noise power is the same both for MBS and FBS receivers. Then, the signal-noise ratio (SNR) received by the *i*-th MUE is expressed as:

(1) $$\begin{array}{c} SNR_{i}=\frac{P_{i}G_{i}}{\sigma^{2}}, \end{array}$$

where $P_{i}$ is the transmission power of the MBS. We assume that a user has fixed transmission power per unit bandwidth. $G_{i}$ is the average channel gain in the current cell.

We define the *i*-th MUE’s spectrum efficiency $\theta_{i}$ according to Equation (1) as follows:

(2) $$\begin{array}{c} \theta_{i}=\log_{2}\left(1+SNR_{i}\right). \end{array}$$

Therefore, the achieved data rate of $MU_{i}$ is $\theta_{i}b$ bits per second by obtaining *b* Hz of spectrum. Note that symbol $X_{i}$ is equivalent to the *i*-th *X* in this paper. We define the data rate threshold as $R_{th}={\left(\theta b\right)}_{th}$. As different MUEs have different channel conditions, they have different achieved data rates even if they use the same amount of bandwidth. As a wide-covering terminology, quality of experience (QoE) has a strong correlation with the underlying QoS in networks [42]. It can be seen that the QoE is increased with spectrum efficiency and the bandwidth getting larger for a certain MUE. Additionally, the greater *θ* and *b* are, the better the QoE is.

## 4. Auction Formulation

In this section, we will discuss the auction framework, including utility functions of MBS and FBS, respectively. From the point of view of economics, auction is a decentralized market mechanism to allocate resources [36]. From the point of view of game theory, both the MBS and FBSs are rational and selfish agents, so that they make all of the possible strategies to increase their own revenue. For the MBS, making profit by the pricing strategy is its main concern. In order to motivate MBS to lease spectrum, FBSs are willing to pay some compensation to rent corresponding bandwidth by their bids.

Before we define the revenue functions of MBS and FBS, we first discuss how to determine the optimal spectrum bandwidth demand of each UE. UEs are the source of revenue for wireless service providers. An UE can acquire data access service only if it gets a certain amount of spectrum.

### 4.1. The Optimal Spectrum Demand of UE

Because UEs need to buy spectrum resources from the service provider, the utility of an UE can be defined as the difference between Experience and Cost, where the Experience is the utility of data rate ln(1+θb) and the Cost is a linear payment for buying *b* Hz spectrum from its service provider, who determines the price *γ*. The utility function *U* of an UE within the coverage of MBS or FBSs can be defined in a common formulation [40] as follows:

(3) $$\begin{array}{c} U=\ln(1+\theta b)-\gamma b, \end{array}$$

where the item γb denotes the payment for resource purchasing to the seller service provider. It is easy to check the first-order derivative of Equation (3). The optimal value of bandwidth demand that maximizes the UE’s utility is:

(4) $$\begin{array}{c} b^{*}=\left\{\begin{array}{cc} \frac{1}{\gamma }-\frac{1}{\theta } & \gamma \leq \theta ,\\ 0 & \gamma >\theta . \end{array}\right. \end{array}$$

**Figure 4:**
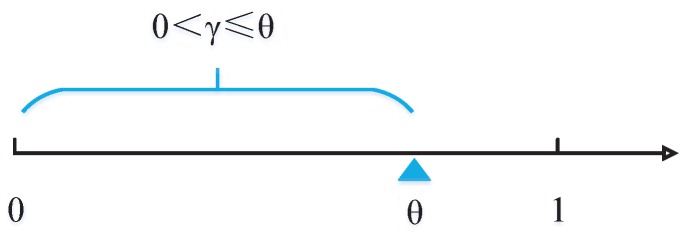
The acceptable service price range for a user equipment (UE) with *θ*.

It can be seen that the optimal spectrum bandwidth decreases with *γ*, but increases with *θ*, with γ≤θ. For a certain UE with spectrum efficiency *θ*, the acceptable service price range is shown as Figure 4. On the one hand, a higher *θ* means that the UE can accept a higher service price, which may bring more revenue for the wireless service provider. On the other hand, the wireless service provider can announce a high service price to filter those UEs with low spectrum efficiency.

### 4.2. The Utility Function of FBS

In closed access mode, each FBS only serves its own subscribers. Due to the FHs owning no spectrum resource, in order to serve FUEs, each FBS $F_{i}$ in the range of the MBS is willing to rent spectrum from the MSP. As buyers, all FBSs who want to obtain spectrum resource will have to participate in the auction. Each FBS decides its rental price $l_{F,M}$ independently for maximizing its own utilities. At the same time, each FBS decides its service price per unit bandwidth as a seller. We define the utility function $U_{i}^{F}$ of the *i*-th FBS as the difference of service revenue $u_{i}^{F}$ and rental cost $c_{i}^{F}$, formulated as follows:

(5) $$\begin{array}{c} U_{i}^{F}=u_{i}^{F}-c_{i}^{F}, \end{array}$$

(6) $$\begin{array}{c} u_{i}^{F}=\sum_{j=1}^{T_{i}}\gamma_{i,j}b_{i,j}, \end{array}$$

where $T_{i}$ is the subscriber set of $F_{i}$ and $\gamma_{i,j}$ denotes the service price of $F_{i}$ serving the *j*-th FUE. $b_{i,j}$ is the required spectrum amount of the *j*-th FUE.

(7) $$\begin{array}{c} c_{i}^{F}=l_{i,M}b_{i,M}, \end{array}$$

where $b_{i,M}$ is the total spectrum requirement of FUEs served by $F_{i}$ and the amount of spectrum that $F_{i}$ would rent from the MSP, as well. Additionally, $l_{i,M}$ is the rent price that $F_{i}$ bids to MBS. Note that the utility function of $F_{i}$ is independent of the others. Each FBS only considers its own pricing strategy.

### 4.3. The Utility Function of MBS

In our model, the MBS is the resource owner that decides which MUEs to serve. For certain MUEs with a bad channel condition, the MBS has to make a low price to meet their spectrum demand, as we analyzed at the end of Section 4.1. However, this pricing strategy tolerates low spectrum efficiency and insufficient resource utilization and results in less revenue of MBS. Therefore, the MBS is willing to lease its spectrum to closer FBSs to improve the spectrum efficiency and its profit. From the perspective of the MBS, leasing spectrum to FBSs would obtain higher profit. That is what the MBS really cares about.

According to the analysis above, we consider the utility function of MBS, which is composed of two parts. One is the reserved revenue of its own MUEs, denoted as $v^{M}$, and the other is the additional revenue of leasing spectrum to FBSs, denoted as $w^{M}$. We formulate it as:

(8) $$\begin{array}{c} U^{M}=v^{M}+w^{M}, \end{array}$$

(9) $$\begin{array}{c} v^{M}=\sum_{i=1}^{K}x_{i}c_{i}^{F}, \end{array}$$

where $x_{i}$ is the spectrum allocation factor and x∈{0,1}. $x_{i}$ = 1 means the spectrum renting demand of FBS *i* is satisfied, and $x_{i}$ = 0 otherwise. The value of $x_{i}$ is determined by the MBS.

(10) $$\begin{array}{c} w^{M}=\sum_{j=1}^{\Gamma }\gamma_{j,M}b_{j,M}, \end{array}$$

where Γ is the set that contains all of the MUEs served by MBS, which is the subset of ${\left\{MU_{i}\right\}}_{i=1}^{L}$. $\gamma_{j,M}$ denotes the service price that MBS serves the *j*-th MUE, and note that $\gamma_{j,M}$ equals $\gamma_{k,M}$ for any j,k∈Γ. Additionally, $b_{j,M}$ represents the spectrum bandwidth demand of the *j*-th MUE.

### 4.4. Network Objective

The spectrum leasing of the femtocell network is motivated by the proposed auction mechanism played by MBS and all FBSs. The objective of MBS is to obtain profit as much as possible by selling the limited spectrum resource. Meanwhile, the objective of the FBS is to serve its subscribers by renting spectrum from the MBS and making a profit. In order to motivate the MBS to lease its resource, compensation is paid by the winner FBSs to the MBS.

For the MBS, there are three options to make a profit: (1) sell all spectrum to its own UEs; (2) sell all spectrum to FBSs; (3) sell part of the spectrum to its own UEs and the others to FBSs. Therefore, the question is how to choose the best option to obtain the maximal profit and how to determine the amount of the “part”.

On the side of FBSs, serving FUEs is their source of revenue. However, they have no resource. In order to get spectrum from the MBS, each FBS *i* in the range of the macrocell submits a bid $l_{i,M}$ to the MBS, which means that FBSs have to split part of their income to the MBS for buying spectrum. To some extent, this situation limits FBSs from achieving the maximal profit.

It can be seen that the market is a monopoly market dominated by the resource owner MBS. As a monopolist, the MBS sets its ultimate goal as maximizing its profit $U^{M}$ via pricing and spectrum allocation. We will discuss the details in the next section.

## 5. Auction-Based Spectrum Leasing Protocol

In this section, we will discuss the spectrum leasing protocol. We pointed out three options for the MBS to obtain profit in Section 4.4. In fact, Option 1 and Option 2 are special cases of Option 3.

**Figure 5:**
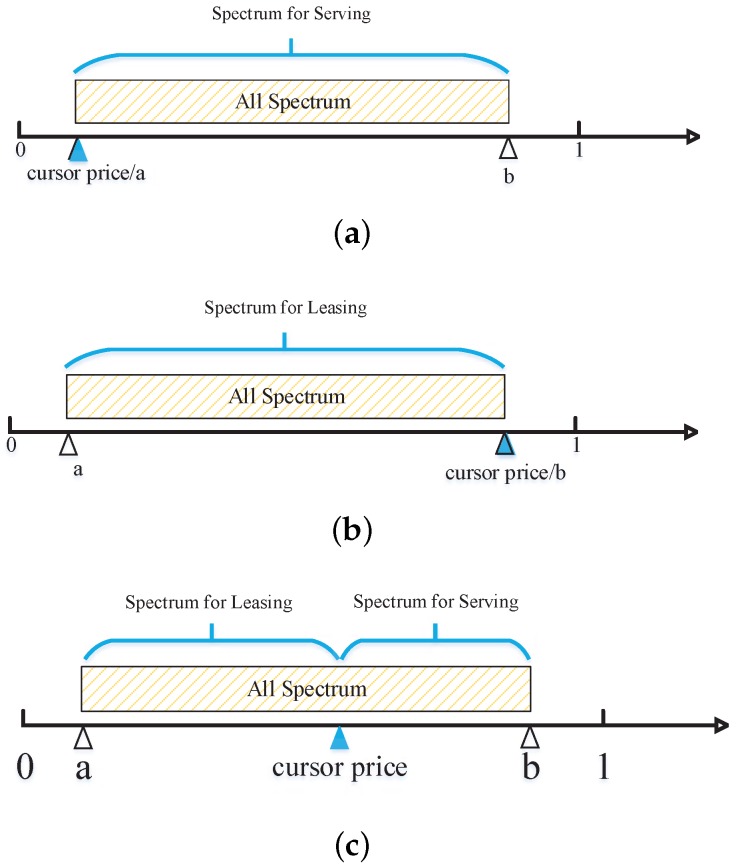
Three options for MBS: the effect of service price on spectrum allocation. (**a**) Option 1: sell all spectrum to MUEs; (**b**) Option 2: sell all spectrum to FBSs; (**c**) Option 3: sell part of the spectrum to MUEs and part to FBSs.

In order to obtain the maximal profit, the MBS has to sell out its spectrum. The effect of macrocell service price on spectrum allocation is shown as Figure 5. There are three important points in the figure, price *a*, price *b* and cursor price.

For Option 1, $w^{M}$ is zero, and the MBS needs to announce a lower price *a* to make the spectrum be sold out exactly, as shown in Figure 5a. At this moment, all spectrum will be used to serve MUEs, and the MBS revenue reaches the maximal value.

Similarly, for Option 2, $v^{M}$ is zero, and the MBS announces a higher price *b*, as shown in Figure 5b. In this price environment, all spectrum will be leased to FBSs. Additionally, the spectrum leasing problem can be modeled as a combinatorial optimization problem. The auctioneer MBS can choose the optimal combination from all bidders to maximize its revenue.

Option 3 is the mixture of the other two, as shown in Figure 5c. At first, the MBS makes a service price, denoted as cursor price, to allow some MUEs with a good channel condition access. Those MUEs would not use up all of the spectrum resources, so that the rest of the spectrum can be used for auction.

Generally, an auction consists of three parts: bidders, an auctioneer and a good for auction. In our model, the MBS owns all of the available spectrum and allocates resource to FBSs; so the bidders are the FBSs, and the auctioneer is the MBS. Obviously, the good for auction is the spectrum resource. Additionally, our auction can be described as follows:

- Bidding: FBSs submit their rental price $L=(l_{1},l_{2}...)$ and bandwidth demand $B=(b_{1},b_{2}..)$ to the MBS. Considering the utility function Equation (9) of $F_{i}$, the larger $l_{i}$ and $b_{i}$ are, the more payment $F_{i}$ pays to MBS. However, even if this $F_{i}$ can bring a high revenue, it will not be guaranteed to win; because the spectrum allocation factor $x_{i}$ is determined by MBS. Therefore, $F_{i}$ has no motivation to lie.
- Allocation: According to the parameters submitted by FBSs, the MBS computes an optimal spectrum allocation $B*=(b_{1}^{*},b_{2}^{*}..)$ that maximizes the revenue by solving a knapsack problem, given the constraints:
  $$\begin{array}{c} \sum B*\leq W. \end{array}$$
- Charging: The MBS computes the payment each FBS should pay according to the spectrum allocation. Note that it is not necessary to order all of the biddings in our auction. For winner $F_{i}$, the payment is $l_{i}*b_{i}$.

We will discuss the auction details in Section 5.1 and Section 5.2. Now, we study how to decide the cursor price to maximize the revenue of the MBS. The leasing protocol among multiple FBSs is carried out in two phases.

1. Each FBS first makes its service price and rental price to the MBS, then it calculates how much spectrum bandwidth needs to be rented, and finally, it submits the rental price and spectrum demand to the MBS.
2. The MBS declares its cursor price $\gamma_{u,M}$ to filter MUEs whose achieved data rate is less than $R_{th}^{M}$. Then, the MBS determines and broadcasts the winner FBSs in the competition and leases spectrum to them.

Note that the rental price of the FBSs should be determined in the first step. If not, the FBSs will have no spectrum to rent. This is because the MBS will allocate all of its spectrum bandwidth to its MUEs to obtain the maximal profit without knowing how much price the FBSs will submit. As a result, there will be no incentive for the MBS to lease its resource.

### 5.1. Femtocell Service Price and Bid Determination

In general, the FUEs are close to FBSs with small path loss. Therefore, the FUEs accessing FBSs will experience superior spectrum efficiency. As a result, FBSs could achieve higher revenue with a higher service price.

Firstly, each FBS collects the channel state information of its subscribers. The performance of those subscribers is usually good enough to provide various services due to the advantage of the small coverage of FBSs. Then, we consider the pricing that can maximize the revenue of FBSs. From the definition of Equation (5), the utility function of each FH *i* is:

$$\begin{array}{cc} & U_{i}^{F}=\sum_{j=1}^{T_{i}}\gamma_{i,j}b_{i,j}-l_{i,M}b_{i,M}\\ & =\sum_{j=1}^{T_{i}}\left(1-\frac{\gamma_{i,j}}{\theta_{i,j}}\right)-\sum_{j=1}^{T_{i}}\left(\frac{l_{i,M}}{\gamma_{i,j}}-\frac{l_{i,M}}{\theta_{i,j}}\right)\\ & =\sum_{j=1}^{T_{i}}\left(1-\frac{\gamma_{i,j}}{\theta_{i,j}}-\frac{l_{i,M}}{\gamma_{i,j}}+\frac{l_{i,M}}{\theta_{i,j}}\right). \end{array}$$

For the FUE *j*, service price $\gamma_{i,j}$ should meet the following equation:

(11) $$\begin{array}{c} {\left\{\gamma_{i,j}\right\}}^{*}=\arg\max\left(1-\frac{\gamma_{i,j}}{\theta_{i,j}}-\frac{l_{i,M}}{\gamma_{i,j}}+\frac{l_{i,M}}{\theta_{i,j}}\right), \end{array}$$

$$\begin{array}{c} s.t.\left\{\begin{array}{c} \gamma_{i,j}>0,\\ \gamma_{i,j}\leq \theta_{i,j}. \end{array}\right. \end{array}$$

By solving the above problem, we can have:

(12) $$\begin{array}{c} {\left\{\gamma_{i,j}\right\}}^{*}=\sqrt{\theta_{i,j}l_{i,M}}. \end{array}$$

The FBS declares different prices for different FUEs, and for a given renting price $l_{i,M}$, the optimal service prices of the FUE *j* are increased with its spectrum efficiency *θ*. Therefore, the FBS’s maximal payoff is:

(13) $$\begin{array}{c} \sum_{j=1}^{T_{i}}\left(1+\frac{l_{i,M}}{\theta_{i,j}}-2\sqrt{\frac{l_{i,M}}{\theta_{i,j}}}\right). \end{array}$$

Note that $0<\sqrt{\theta_{i,j}l_{i,M}}\leq \theta_{i,j}$, then $0<l_{i,M}\leq \theta_{i,j}$.

Now, we consider how to decide the rental price. It can be seen that the maximal payoff decreases with the increase of rental price between zero and $\theta_{i,j}$, which means that if $l_{i,M}=0$, the FBS can obtain maximal profit. However, the MBS will not lease spectrum to an FBS with zero profit. As a bidder, the FBS has to improve its rental bid for winning the competition. On the other hand, if $l_{i,M}=\theta_{i,j}$, the revenue of serving the *j*-th FUE is zero. In order to protect the FBS, we have this equation:

(14) $$\begin{array}{c} l_{i,M}=\min\theta_{i,j}-\delta_{i}, \end{array}$$

where $\delta_{i}$ is the reserve price set by $F_{i}$, $0\leq \delta_{i}<\min\left\{\theta_{i,j}\right\}$ to ensure the FBS achieves a non-negative profit. At the same time, the spectrum demand of $F_{i}$ is:

(15) $$\begin{array}{c} b_{i,M}=\sum_{j=1}^{T_{i}}\left(\frac{1}{\gamma_{i,j}}-\frac{1}{\theta_{i,j}}\right). \end{array}$$

Then, the FBS submits $l_{i,M}$ and $b_{i,M}$ to the MBS.

### 5.2. Macrocell Service Price and Winner Determination

Now, we discuss how to make the cursor price for the MBS. To achieve the threshold $R_{th}^{M}$, the spectrum demand of MUEs must be larger than $\frac{R_{th}^{M}}{\theta }$. Since the optimal value of *b* is $\frac{1}{\gamma }-\frac{1}{\theta }$ according to Equation (3), we can know that:

$$\begin{array}{c} \frac{R_{th}^{M}}{\theta }\leq \frac{1}{\gamma }-\frac{1}{\theta }. \end{array}$$

Then, we can have:

(16) $$\begin{array}{c} \gamma \leq \frac{\theta }{R_{th}^{M}+1}. \end{array}$$

We define $\gamma_{th}^{i,M}=\frac{\theta_{i}}{R_{th}^{M}+1}$ as the critical price of the MUE *i*, which means that the achieved data rate of the MUE *i* is not less than the rate threshold if the cursor price is not more than $\gamma_{th}^{i,M}$.

Obviously, we have the critical price set ${\left\{\gamma_{th}^{i,M}\right\}}_{i=1}^{L}=\left\{\gamma_{th}^{1,M},\gamma_{th}^{2,M}\cdots \gamma_{th}^{L,M}\right\}$ for all MUEs. We consider that all MUEs adopt the same service price, and then, for a certain cursor price, those MUEs whose critical price is less than the service price will be out of service, and then, the MBS leases its spectrum to FBSs for auction. Now, we discuss how the MBS chooses the winner FBSs.

The utility of the MBS is:

(17) $$\begin{array}{c} U^{M}=\sum_{i=1}^{K}x_{i}l_{i,M}b_{i,M}+\sum_{j=1}^{\Gamma }\gamma_{j,M}b_{j,M}, \end{array}$$

The second item in Equation (17) is fixed for a given cursor price $\gamma_{j,M}$, so that the MBS determines the winner(s) by maximizing the leasing revenue $\Sigma_{i=1}^{K}x_{i}l_{i,M}b_{i,M}$, which can be modeled as a 0–1 knapsack problem. The formulation of the problem is given by:

(18) $$\begin{array}{c} \max\sum_{i=1}^{K}x_{i}c_{i}^{F}, \end{array}$$

$$\begin{array}{c} s.t.\left\{\begin{array}{c} \sum_{i=1}^{K}x_{i}b_{i,M}\leq W^{\prime },\\ x_{i}=0\;or\;1, \end{array}\right. \end{array}$$

where $c_{i}^{F}$ and $b_{i,M}$ mean the value and the weight in the knapsack problem, respectively. For a certain price $\gamma_{j,M}$, the spectrum that has been used is $\sum_{j=1}^{\Gamma }\left(1-\frac{\gamma_{j,M}}{\theta_{j,M}}\right)$; then, the rest of spectrum, i.e., knapsack capacity, is:

(19) $$\begin{array}{c} W^{\prime }=W-\sum_{j=1}^{\Gamma }\left(1-\frac{\gamma_{j,M}}{\theta_{j,M}}\right). \end{array}$$

We will present the knapsack algorithm and winner determination algorithm in the next subsection.

### 5.3. Algorithm Design

According to the above subsection, the spectrum leasing algorithm consists of two parts: the combinatorial problem and cursor price determination strategy. Next, we design an algorithm for each part.

The 0–1 knapsack problem can be solved by the greedy algorithm or the dynamic programming algorithm (DPA), and both of the two strategies exploit the optimal substructure. However, the greedy algorithm cannot guarantee having an optimal solution; because it depends on the condition of data. Differently, dynamic programming is known as a solution to a variety of optimization problems, and it works toward decomposing a big problem into a sequence of smaller sub-problems, solving these sub-problems and storing their optimal values using matrix structures [43]. The DPA must be able to get an optimal solution for the 0–1 knapsack problem. Additionally, it has the advantage of lower computational complexity.

We denote the bandwidth demand array as *B*, where $B=[b_{1,M},b_{2,M},...,b_{K,M}]$. Among them, $B\left[i\right]=b_{i,M}$ represents the spectrum demand of the *i*-th FBS. Denote the rental fee array as *V*, where $V=[l_{1,M}*b_{1,M},l_{2,M}*b_{2,M},...,l_{K,M}*b_{K,M}]$. Additionally, $V\left[i\right]=l_{i,M}*b_{i,M}$ represents the rental fee of the *i*-th FBS. Denote the rest of current available spectrum as *n*. Additionally, we define a two-dimensional array Rev[][] to store each sub-problem’s optimal values. Rev[i][n] represents the leasing revenue of MBS after the MBS leased spectrum to the *i*-th FBS. Note that whether the $F_{i}$ can obtain the spectrum resource depends on the MBS.

Now, we discuss the condition of revenue. Assume that the current revenue of the MBS is Rev[i][n] after it selected the *i*-th FBS. At this time, the MBS has two choices when the spectrum demand of $F_{i+1}$ is less than the rest *n*. One is leasing spectrum to it with revenue Rev[i][n−B[i]]+V[i]. The other is not leasing spectrum to it with revenue Rev[i][n]. Otherwise, the MBS has only one choice, which is not leasing spectrum to $F_{i+1}$ with revenue Rev[i][n].

Therefore, we design a knapsack algorithm based on generalized dynamic programming by analyzing the recursion Formula (20).

(20) $$\begin{array}{cc} & Rev\left[i+1\right]\left[n\right]=\left\{\begin{array}{cc} \max(Rev[i][n],Rev[i][n-B[i]]+V[i]) & n\geq B[i],\\ Rev[i][n] & otherwise, \end{array}\right. \end{array}$$

where i∈[1,K] and Rev[0][n]=0 for any n∈[0,W]. Note that the array index must be a positive integer, while *n* may be a non-integer. To reduce this error, we magnify *n* one thousand times and take the integer. Lastly, we shrink the result value one thousand times. Meanwhile, we define a two-dimensional Boolean array path[][] to store the winner FBS index, where path[i][n]∈{0,1}.

The details of the knapsack process are shown in Algorithm 1. At the beginning of Algorithm 1, we create two two-dimensional arrays $Rev\left[N\right]\left[W^{\prime }\right]$ and $path\left[N\right]\left[W^{\prime }\right]$ and fill them with zero. Lines 3 to 19 are used to calculate all of the sub-problems and store their optimal values. Just iterate all of the FBSs. If the higher revenue can be obtained by leasing spectrum to $F_{i}$, then lease to it, otherwise do not lease to it. In Line 11, the $F_{i+1}$ get the spectrum resource, then the path[i+1][n] is set to one. Lines 20 to 26 are used to select the winner FBSs. Iterate array $path\left[N\right]\left[W^{\prime }\right]$, and put the $F_{i}$ into knapsack *k* when path[i][n] equals one.

**Algorithm 1** Dynamic programming-based knapsack algorithm.**Input:** FBS count *N*, $W^{\prime }$, *B*, *V*, current knapsack index *k***Output:** Max revenue res1:Initial: Fill the two-dimensional matrix $Rev\left[N\right]\left[W^{\prime }\right]$ and $path\left[N\right]\left[W^{\prime }\right]$ with 02:i=0, n=03:**while**
i<N
**do**4:    n=0;5:    **while**
$n\leq W^{\prime }$
**do**6:        **if**
n<B[i]
**then**7:           Rev[i+1][n]=Rev[i][n];8:        **else**9:           **if**
Rev[i][n]<Rev[i][n−B[i]]+V[i]
**then**
10:               Rev[i+1][n]=Rev[i][n−B[i]]+V[i];
11:               path[i+1][n]=1;12:           **else**13:               Rev[i+1][n]=Rev[i][n];14:           **end**
**if**15:        **end**
**if**16:        n=n+1;17:    **end**
**while**18:    i=i+1;19:**end**
**while**20:**while**
i≥0andn≥0
**do**21:    **if**
path[i][n]==1
**then**22:        put
*i* into knapsack *k*;23:        n=n−B[i];24:    **end**
**if**25:    i=i−1;26:**end**
**while**27:$res=Rev\left[N\right]\left[W^{\prime }\right]$;

Here, we analyze the time complexity of Algorithm 1. At the beginning of the program, the cost of initialization and assignment operations can be ignored. We mainly analyze the time complexity of the knapsack process, i.e., Lines 3 to 26 of Algorithm 1. There is a dual-layer iteration in Lines 3 to 19. The inner-most loop will run $W^{\prime }$ times; meanwhile, the outer-most loop will run *N* times. Therefore, Lines 3 to 19 consume time $T_{1}$, which is $N*W^{\prime }$. Similarly, Lines 20 to 26 need time $T_{2}$, which is $\min\{N,W^{\prime }\}$. In summary, the total time cost of the knapsack process *T* is $T_{1}+T_{2}=N*W^{\prime }+\min\left\{N,W^{\prime }\right\}$. Then, the time complexity of Algorithm 1 is $O(NW^{\prime })$.

Then, we solved the problem (18) via Algorithm 1. Therefore, the problem (17) can be solved by iterating all possible macrocell service prices. We designed a price and winner determination algorithm to obtain the max revenue and the optimal service price of the MBS. At the same time, the MBS determines the winner FBSs and leases spectrum to them.

As shown in Algorithm 2, we first initialize the cursor price *γ*, optimal knapsack index *k*, current max revenue MR and current price OP. Then we try to iterate all the possible service price with the step of 0.01 (Lines 5 to 19). For a certain price, the revenue of serving MUEs and the usage amount of spectrum can be calculated, denoted as $R_{1}$ and $W_{1}$, respectively, as shown in Line 6. Therefore, the rest of available spectrum $W^{\prime }$ is $W-W_{1}$ in Line 7. Lines 8 to 16 are used to calculate the leasing revenue $R_{2}$, which can be obtained by Algorithm 1. Therefore, the total revenue of MBS is $R=R_{1}+R_{2}$. If the *R* is greater than the current max revenue, we update the max revenue MR and the corresponding price OP. Repeat the process until we find the optimal revenue. Finally, lease spectrum to winner FBSs in knapsack *k* in Line 22.

**Algorithm 2** Price and winner determination algorithm.**Input:**
*W*, *B*, *V*, MUE count *L*, FBS count *K***Output:** cursor price *γ*, $U^{M}$1:Initial: γ=0.01, i=12:Initial: optimal knapsack index k=−13:Initial: current max revenue MR=04:Initial: current price related with max revenue OP=05:**while**
γ<1
**do**6:    Calculate the reserved revenue $R_{1}$ and bandwidth usage $W_{1}$;7:    Calculate the available leasing bandwidth $W^{\prime }=W-W_{1}$;8:    **if**
$W^{\prime }>0$
**then**9:        Calculate the leasing revenue $R_{2}$ via Algorithm 1 by inputting *K*, $W^{\prime }$, *B*, *V*, *i*;10:        Calculate the total revenue $R=R_{1}+R_{2}$;11:        **if**
MR<R
**then**12:           MR=R;13:           OP=γ;14:           k=i;15:        **end**
**if**16:    **end**
**if**17:    γ=γ+0.01;18:    i=i+1;19:**end**
**while**20:$U^{M}=MR$;21:γ=OP;22:Lease spectrum to these FBSs in knapsack *k*;

Now, we discuss the time complexity of Algorithm 2. We mainly analyze the time cost of the loop in Lines 5 to 19, which will run $\frac{1}{\gamma }$ times. $\frac{1}{\gamma }$ can be denoted as *T*. Among them, Line 6 consumes time *L* to iterate all MUEs and calculate serving revenue. According to Algorithm 1, Line 9 consumes time $K*W^{\prime }$. We can conclude that the total time cost of Algorithm 2 is $T(L+NW^{\prime })$. Therefore, the time complexity of Algorithm 2 is $O(TNW^{\prime })$.

#### Optimality of Dynamic Programming

A problem that can be solved by dynamic programming must have two main properties: optimal substructures and overlapping sub-problems. For a problem of size *n*, the optimal solution is based on optimal solutions to the same sub-problems. In this paper, the knapsack problem can be split into smaller problems. Then, we need only to consider the optimal solutions to sub-problems instead of all possible solutions. As we discussed in Equation (20), Rev[i+1][n]=max(Rev[i][n],Rev[i][n−B[i]]+V[i]), the optimal substructure is Rev[i+1][n]. It can check only the optimal solution to Rev[i][n], and non-optimal solutions are not considered.

In the next section, the simulation results will show that our pricing strategy can help the MBS achieve more profit.

## 6. Simulation Results

In this section, we conduct numerous simulations using MATLAB to show the outcome of the proposed framework. We consider a two-tier network where tens of femtocells exist within the coverage of the macrocell providing services to a certain number of users in a certain geographic area.

### 6.1. Effect on Incentives

**Figure 6:**
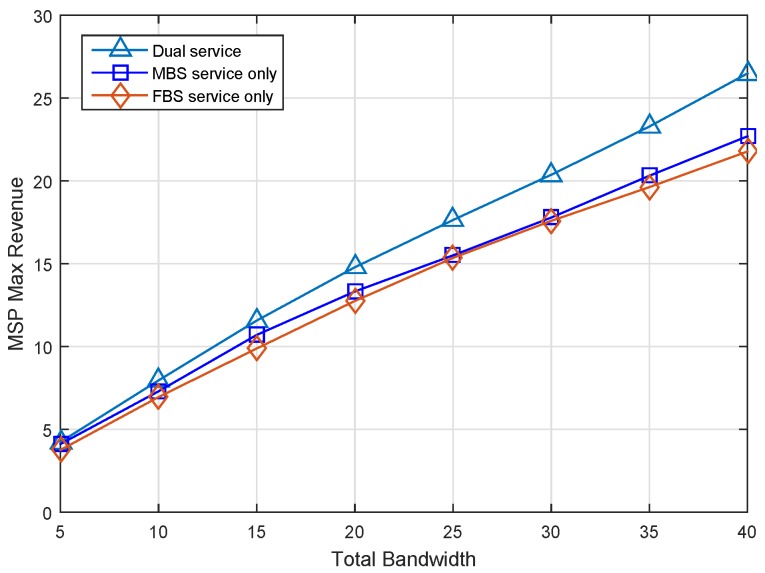
Macrocell service provider (MSP) max revenue versus total bandwidth under different service modes.

We first investigate the revenue condition of the MBS who owns the total available spectrum bandwidth from 5 MHz to 40 MHz with the step 5 MHz. Assume that there are 200 FBSs and 200 MUEs in the scenario, and we set the data rate threshold as 0.1. Then, we simulate the MSP max revenue under different service modes as illustrated in Figure 6. In our scenario, the spectrum efficiency of MUEs can be normalized in the range [0,1] (i.e., θ∈ [0,1]), and we assume *θ* is uniformly distributed. The FUEs achieve high spectrum efficiency due to the advantages of the femtocell. In MBS service-only and FBS service-only modes, the data access service will only be provided by the MBS and FBSs, respectively. Otherwise, the access service will be provided by both the MBS and FBSs.

It can been see that the max revenue of MSP in dual service mode is always greater than the other two modes in the same amount of bandwidth in Figure 6. In the mode of FBS service only, the MSP obtains the lowest revenue by leasing all spectrum resources to FBSs without serving its own MUEs. This is because a part of the whole revenue of the MSP will be split to FHs, which results in decreased profit of the MSP. In the MBS service-only case, the MBS have to tolerate some MUEs with lower spectrum efficiency and make a low service price for them. However, in the dual service case, the MBS would lease the spectrum of those MUEs to FBSs by charging a high leasing price. Obviously, the MSP can obtain optimal revenue in dual service, which means that it is effective to improve the MSP profit by spectrum leasing. Additionally, the simulation results show that the more bandwidth, the more obvious the improvement of the revenue.

### 6.2. Impact of Spectrum Bandwidth

**Figure 7:**
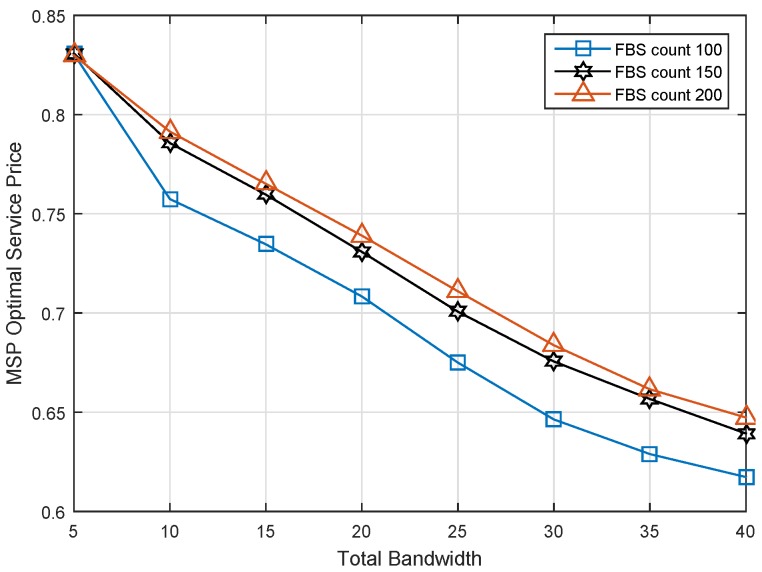
Optimal service price versus total bandwidth under different FBS counts.

The relationship of total bandwidth and optimal service price is shown as Figure 7. For the MSP, the optimal decision for obtaining the maximal revenue is selling out all of the spectrum resource. When the MSP owns much more spectrum, it would like to make a lower service price to sell them all, resulting in the decrease of service price, but the increase of revenue. On the other hand, in the situation of the same bandwidth, we found that the more the FBS count, the higher the service price. The reason is that many FBSs are more likely to compose the better bids for the MSP. Then, the MSP needs a higher service price to release more spectrum for leasing.

**Figure 8:**
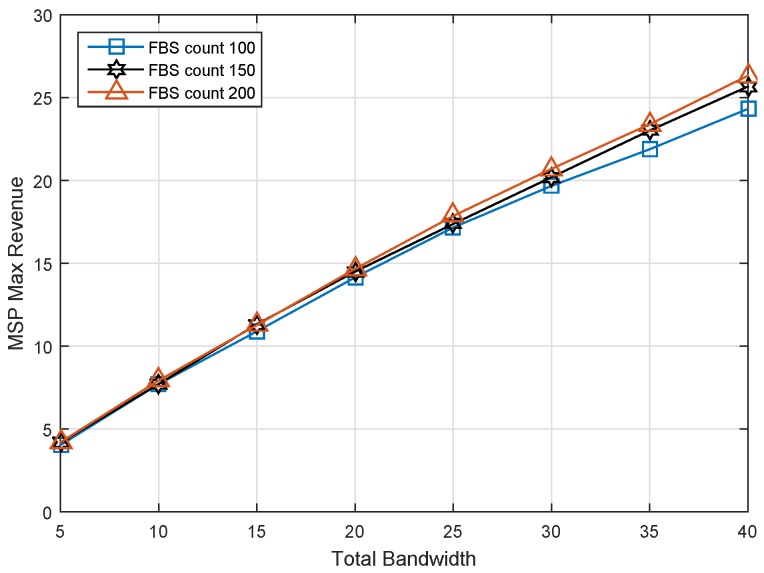
Max revenue of the MSP versus total bandwidth under different FBS counts.

It is noted that three service prices are equal for these three cases at the first point in Figure 7. To investigate the reason for this exception, we studied the revenue of MSP under different FBS counts, and the result is shown as Figure 8. As we can see, the revenues of MSP are the same values when the total spectrum bandwidth is 5 MHz. There was one inference to be drawn from the exception. It is that all of the available spectrum was used by MUEs with the best channel condition. No spectrum could be released for leasing no matter how many FBSs there are.

### 6.3. Impact of MUE Density

**Figure 9:**
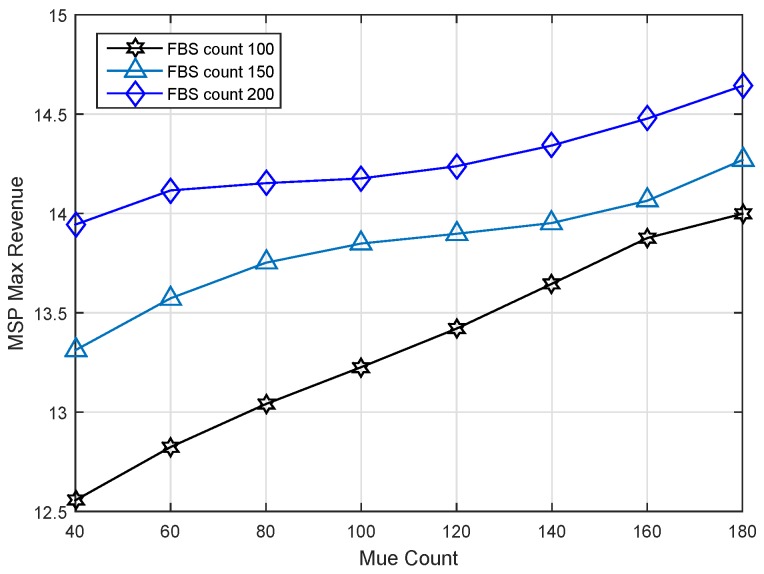
MSP max revenue versus MUE count under different FBS counts.

Moreover, we do a series of experiments to illustrate the impact of MUE density on the results of our strategy. All evaluations are done under the same bandwidth W=20 MHz. Figure 9 demonstrates the impact of MUE density on the revenue of MSP. We can see that the max revenue of the MSP increases with MUE density under different FBS counts; because there are more MUEs with high spectrum efficiency in the case of high MUE density. Similarly, in the situation of the same MUE density, such as 100, the competition among FBSs gets more intense and results in a higher revenue for the MSP.

**Figure 10:**
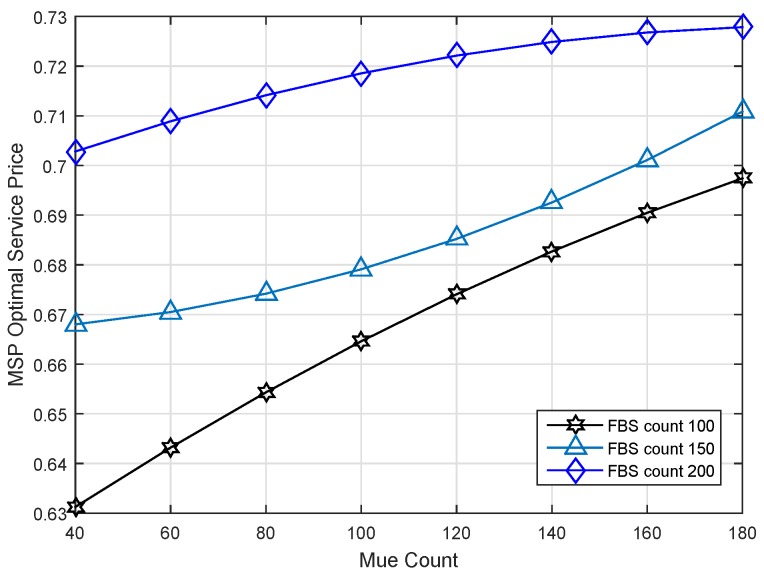
MSP optimal service price versus MUE count under different FBS counts.

As shown in Figure 10, the relationship of MUE count and optimal service price can be presented clearly. As expected, the service price generated is greater when there are more MUE buyers. MUEs are the revenue source of the MSP; it is a benefit for the MSP to attract many MUEs to participate in the competition. There are more MUEs who can accept a higher service price. Therefore, the service price is proportional to the increase of the MUE count.

### 6.4. Impact of FBS Reserve Price

**Figure 11:**
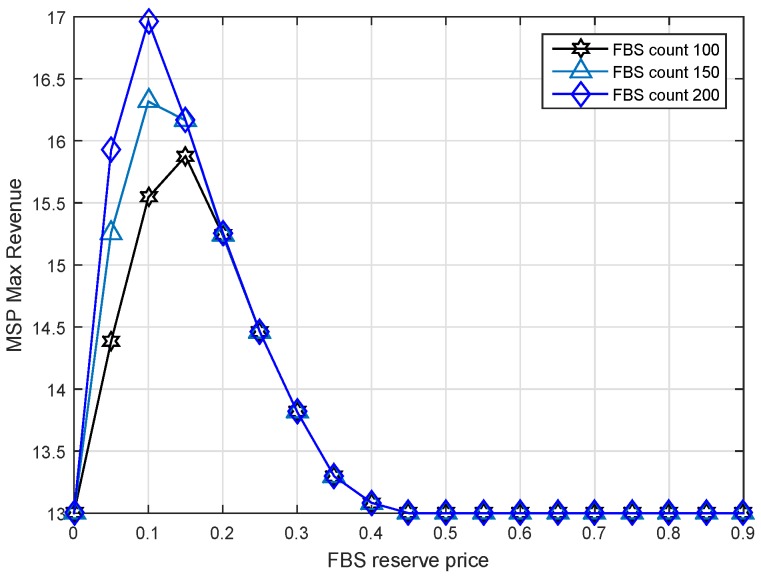
Max revenue of MSP versus FBS reserve price under different FBS counts.

**Figure 12:**
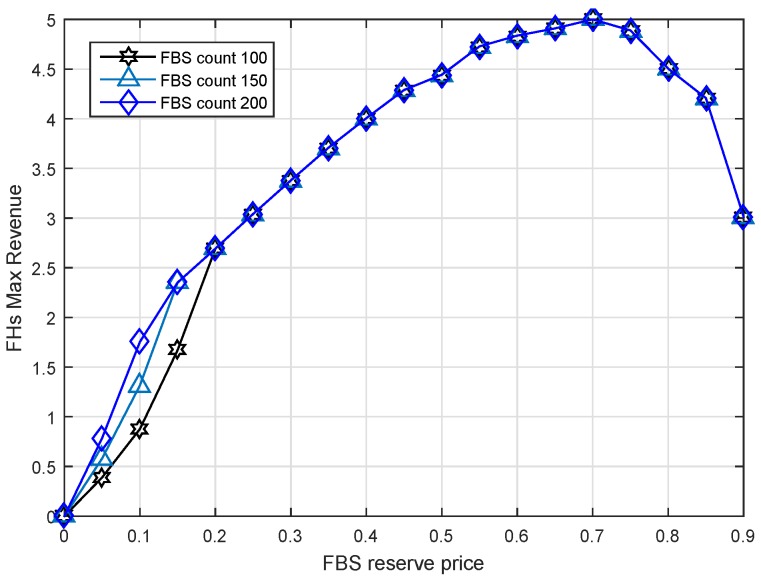
The max revenue of FHs versus FBS reserve price under different FBS counts.

From the point of view of FBSs, they want to know how to bid to improve the possibility of winning. In order to get the answer, we assume that all FBSs determine the same reserve price ranging from 0 to 0.9 with the step of 0.05. There were two experiments done to show the results. We show the revenue condition of the MSP in Figure 11 against the FBS reserve price. Additionally, the revenue condition of FHs is shown in Figure 12.

We found that when the reserve prices are distributed between 0.05 and 0.45, the MSP max revenue reaches a higher level, as shown in Figure 11. However, the FHs would not obtain the max profit at this time as shown in Figure 12. In fact, if the FHs set their reserve price greater than 0.45 for obtaining larger revenue, the MSP would lease zero spectrum to them in order to maintain its own benefit. Because the market is a monopoly market dominated by the MSP. It can be seen that the market mechanism protects the FHs profit that is greater than zero, but it also limits them from achieving the maximal profit.

Note that the max revenue is increased first and then decreased in Figure 11. At the first point, FBS reserve price zero means the FHs will hand over all of their serving income to the MSP. It seems that the MSP should obtain the maximal revenue rather than the minimum. However, when the FBS reserve price is set to zero, the service price of the FBS will be unaffordable for FUEs, so that the income of FHs becomes zero. Therefore, the revenue of 13 of MSP is all from serving MUEs without leasing. Similarly, the higher reserve price causes more income to be retained by FBSs themselves so that the MSP would like to sell spectrum to MUEs instead of FBSs as shown after the point of 0.45.

Combining the conclusions of Figure 11 and Figure 12, we can get that those FBSs whose reserve price in the range of 0.05 to 0.45 would obtain a greater probability of winning.

**Figure 13:**
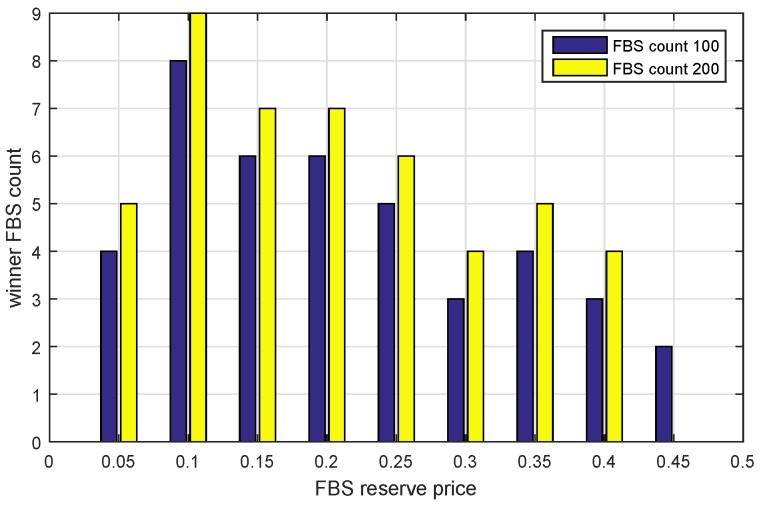
The winner FBS Count distribution under different FBS counts.

To prove that, we made an extra simulation, where all FBSs determine their reserve price randomly. Then, we collect the winner FBSs’ distribution, as shown in Figure 13. The results are in accordance with what we expected. All of the winner FBSs’ distribution ranges from 0.05 to 0.45. Among them, there is a larger amount of FBSs at reserve price 0.1, rather than the other reserve price. This is also in agreement with the results of Figure 11. It is worth noting that reserve price is not the only factor that affects the auction result. The result is also related to the demand of bandwidth. Therefore, although 0.1 is the point that has greater probability of winning, it does not mean that 0.1 is the only point that can win the auction. Therefore, there are multiple maximum points in Figure 13.

### 6.5. Performance of the Network

**Figure 14:**
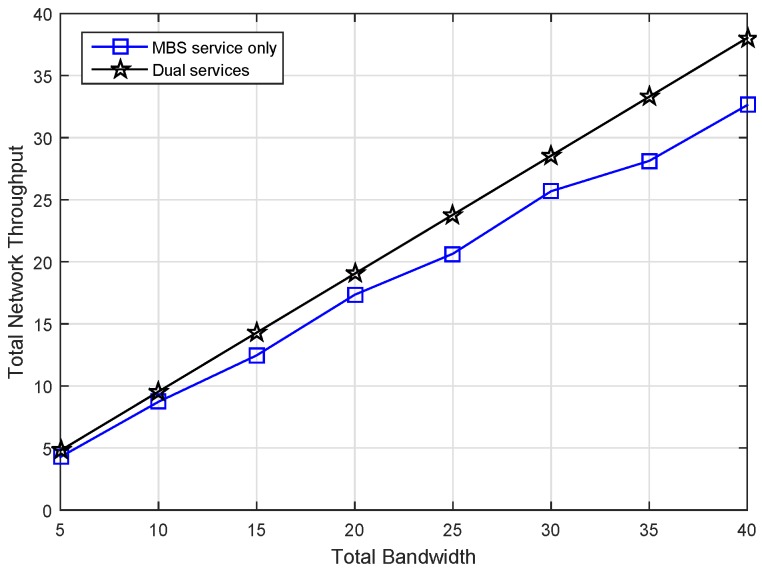
Overall network throughput under different service modes.

As an important QoS metric, throughput has been evaluated in the model. Additionally, we simulate the overall network throughput under different service modes, as shown in Figure 14. As expected, the throughput of dual service mode is always larger than the MBS service only in the same amount of bandwidth. The reason is that in order to lease spectrum to FBSs, the MBS releases some MUEs with low spectrum efficiency. Therefore, the throughput has been significantly improved, as well as the revenue.

## 7. Conclusions

In this paper, we proposed a pricing framework to investigate the spectrum leasing problem for coexisting MBS and FBSs in mobile macro-femto networks. We model the leasing procedure between the MBS and FBSs as an auction, where all FBSs bid to obtain the spectrum resource, and the MBS determines the spectrum leasing amount and chooses the winner FBSs, which maximizes the utilities of the MBS. In order to motivate the MBS to sell resources to the FBSs, we designed a pricing strategy to guarantee the optimal revenue of MSP in the monopoly market. This pricing strategy can be achieved by the knapsack algorithm with lower computational complexity. Finally, the simulation results show that the proposed framework provides effective incentive for the MBS to lease spectrum to FBSs, and both the MSP and FHs can achieve higher utility with spectrum leasing. At the same time, the overall network throughput can be improved significantly.

With the progress of 5G networks, more applications with higher performance requirements, such as IoT and e-health, should be supported. It can be seen that the IoT is going to be considered as a meaningful superaddition to the mobile Internet, while the QoS differentiation and management complication become the key challenges. To satisfy these requirements, other spectrum pricing models for different types of IoT services will be considered in future work.
